# An Overview of High-*k* Oxides on Hydrogenated-Diamond for Metal-Oxide-Semiconductor Capacitors and Field-Effect Transistors

**DOI:** 10.3390/s18061813

**Published:** 2018-06-04

**Authors:** Jiangwei Liu, Yasuo Koide

**Affiliations:** 1 Research Center for Functional Materials, National Institute for Materials Science (NIMS), 1-1 Namiki, Tsukuba 305-0044, Ibaraki, Japan; 2 Research Network and Facility Services Division, National Institute for Materials Science (NIMS), 1-2-1 Sengen, Tsukuba 305-0047, Ibaraki, Japan; koide.yasuo@nims.go.jp

**Keywords:** diamond, high-*k*, MOS capacitor, MOSFET, gas sensors

## Abstract

Thanks to its excellent intrinsic properties, diamond is promising for applications of high-power electronic devices, ultraviolet detectors, biosensors, high-temperature tolerant gas sensors, etc. Here, an overview of high-*k* oxides on hydrogenated-diamond (H-diamond) for metal-oxide-semiconductor (MOS) capacitors and MOS field-effect transistors (MOSFETs) is demonstrated. Fabrication routines for the H-diamond MOS capacitors and MOSFETs, band configurations of oxide/H-diamond heterointerfaces, and electrical properties of the MOS and MOSFETs are summarized and discussed. High-*k* oxide insulators are deposited using atomic layer deposition (ALD) and sputtering deposition (SD) techniques. Electrical properties of the H-diamond MOS capacitors with high-*k* oxides of ALD-Al_2_O_3_, ALD-HfO_2_, ALD-HfO_2_/ALD-Al_2_O_3_ multilayer, SD-HfO_2_/ALD-HfO_2_ bilayer, SD-TiO_2_/ALD-Al_2_O_3_ bilayer, and ALD-TiO_2_/ALD-Al_2_O_3_ bilayer are discussed. Analyses for capacitance-voltage characteristics of them show that there are low fixed and trapped charge densities for the ALD-Al_2_O_3_/H-diamond and SD-HfO_2_/ALD-HfO_2_/H-diamond MOS capacitors. The *k* value of 27.2 for the ALD-TiO_2_/ALD-Al_2_O_3_ bilayer is larger than those of the other oxide insulators. Drain-source current versus voltage curves show distinct pitch-off and *p*-type channel characteristics for the ALD-Al_2_O_3_/H-diamond, SD-HfO_2_/ALD-HfO_2_/H-diamond, and ALD-TiO_2_/ALD-Al_2_O_3_/H-diamond MOSFETs. Understanding of fabrication routines and electrical properties for the high-*k* oxide/H-diamond MOS electronic devices is meaningful for the fabrication of high-performance H-diamond MOS capacitor and MOSFET gas sensors.

## 1. Introduction

Due to limitation of its bandgap energy, thermal conductivity, and electron saturation velocity, Si-based electronic devices cannot meet future demands in fields of high-power, high-temperature, high-frequency, and low power loss. Wide bandgap semiconductors such as SiC, GaN, and diamond are developed to partly replace Si for next-generation power electronic devices [1,2,3]. Table 1 summarizes basic physical properties of Si, 4H-SiC, GaN, and diamond [4,5,6]. Comparing with other semiconductors in this table, diamond has the widest bandgap energy, the highest breakdown field, the largest thermal conductivity, and the largest carrier mobility. Therefore, diamond-based electronic devices are promising for the future applications in fields of high-power handling, high-temperature operation, and high-frequency switching. Meanwhile, since diamond has good chemical inertness, good biocompatibility, and a large electrochemical window, it is also a suitable candidate for applications of biosensors [7,8,9]. Additionally, diamond can also be applied in fields of ultraviolet (UV) light-emitting diodes [10], UV detectors [11], and high-temperature tolerant gas sensors [12,13,14].

Although semiconductor diamond-based devices have many potential applications, lack of shallow dopants hinders the development of them. Activation energies of boron and phosphorus doped *p*-type and *n*-type diamond are as large as 370 and 570 meV at room temperature (RT), respectively. They are much higher than the RT thermal energy of around 26 meV. Although thin heavily boron-doped diamond channel layer is promising to resolve this issue [15,16], its hole mobility is not high and growth technique still needs to be improved. A *p*-type hydrogenated diamond (H-diamond) channel layer is considered as another candidate to resolve this issue [17,18]. Two-dimensional hole gases are accumulated on the surface of the H-diamond caused by transfer of electrons from H-diamond to negatively surface adsorbate layer [19]. Its sheet hole density is around ~10^13^ cm^−2^. After exposing H-diamond in NO_2_ ambient or annealing treatment in NH_3_ + H_2_ ambient, its hole density can be enhanced to be as high as ~10^14^ cm^−2^ [20,21].

Si-, GaAs- and SiC-based metal-oxide-semiconductor (MOS) capacitor and MOS field-effect transistor (MOSFET) gas sensors have been developed greatly [22,23,24,25,26]. Gate metals (such as Pd, Pt, and Ir) have catalytic properties. They can adsorb hydrogen, ammonia, and carbon monoxide gases, leading to the generation of charges at metal/oxide interfaces, which make capacitance-voltage (*C*-*V*) curve shift in the depletion region for MOS capacitors and threshold voltage (*V_TH_*) shift for the MOSFETs [27]. Although diamond has superior properties over other semiconductors, there are rarely reports for the diamond-based MOS capacitor and MOSFET gas sensors. In order to fabricate them successfully, it is important to know the fabrication routines and electrical properties of diamond-based MOS capacitors and MOSFETs.

Recently, fabrication techniques for the H-diamond-based MOS capacitors and MOSFETs have been developed greatly. The electrical properties of them have also been improved. The H-diamond MOS capacitors with low leakage current and trapped charge densities were fabricated [28,29,30,31,32,33,34]. By improving the device structures, T-type and triple-gate fin-type H-diamond MOSFETs were fabricated successfully with current outputs more than 200 mA·mm^−1^ [32,33,34]. The NO_2_-treated H-diamond channel layer based MOSFETs could operate well with a current output as high as 1.35 A·mm^−1^ [35]. Improvement of deposition conditions for the oxide insulators enhanced H-diamond MOSFET’s operation temperature and breakdown voltage to be more than 400 °C and 1000 V, respectively [36]. Additionally, enhancement-mode H-diamond MOSFETs were developed for low power consumption [37,38,39].

**Figure 1:**
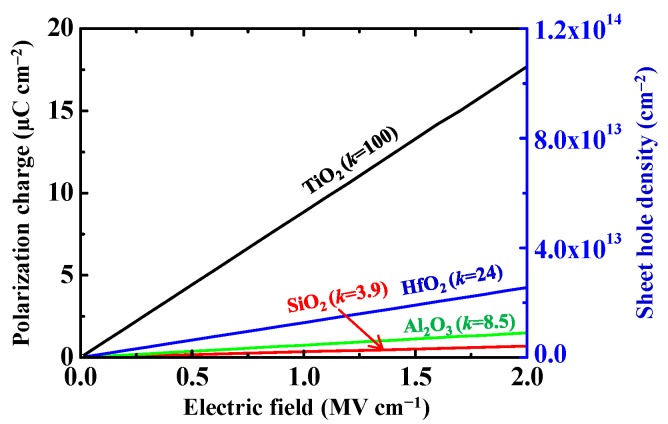
Polarization charge and sheet hole density in the H-diamond as functions of electric field (Reprinted from reference [40]).

Previously, we have focused on fabrication of high-*k* oxide/H-diamond MOS electronic devices [28,29,30,31,32,33]. Figure 1 shows polarization charge and sheet hole density in the H-diamond as functions of electric field [40,41], which is applied to gate oxide insulators of SiO_2_, Al_2_O_3_, HfO_2_ [42], and TiO_2_ [43]. At the same electric field, the oxide insulator with a higher-*k* value can response a larger sheet hole density for the MOS electronic devices. A gate oxide insulator with *k* value around 100 based on TiO_2_ is essential to attain hole densities around 10^14^ cm^−2^ for the H-diamond channel layer. Here, we summarize our previous reports for the Al_2_O_3_, HfO_2_, and TiO_2_ high-*k* oxide insulators on the H-diamond for the MOS electronic devices [28,29,30,31,44]. The oxide insulators are deposited using atomic layer deposition (ALD) and sputtering deposition (SD) techniques. Band configurations of ALD-Al_2_O_3_/H-diamond, ALD-HfO_2_/H-diamond, and ALD-TiO_2_/ALD-Al_2_O_3_/H-diamond heterojunctions are demonstrated. Electrical properties of H-diamond MOS capacitors with oxides of ALD-Al_2_O_3_ [28], ALD-HfO_2_ [29], ALD-HfO_2_/ALD-Al_2_O_3_ multilayer [28], SD-HfO_2_/ALD-HfO_2_ bilayer [30], SD-TiO_2_/ALD-Al_2_O_3_ bilayer [31], and ALD-TiO_2_/ALD-Al_2_O_3_ bilayer [31] are summarized and discussed. Analyses for the *C*-*V* characteristics of them show that there are low fixed and trapped charge densities for the ALD-Al_2_O_3_/H-diamond and SD-HfO_2_/ALD-HfO_2_/H-diamond MOS capacitors. The *k* value of ALD-TiO_2_/ALD-Al_2_O_3_ bilayer is larger than those of other oxide insulators. There are excellent electrical properties for the ALD-Al_2_O_3_/H-diamond, SD-HfO_2_/ALD-HfO_2_/H-diamond, and ALD-TiO_2_/ALD-Al_2_O_3_/H-diamond MOSFETs.

## 2. Materials and Methods

**Figure 2:**
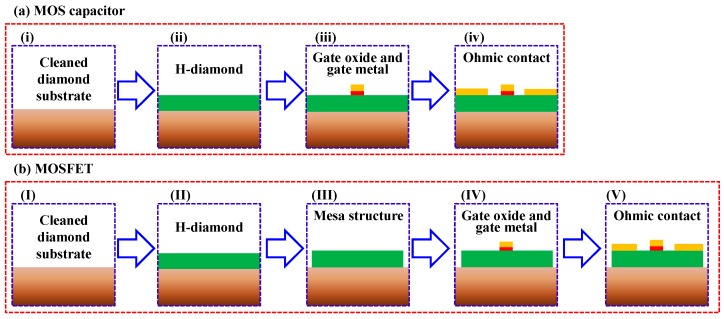
Fabrication routines for H-diamond (**a**) metal-oxide-semiconductor (MOS) capacitor and (**b**) MOS field-effect transistor (MOSFET), respectively.

Figure 2a,b show fabrication routines for the H-diamond MOS capacitor and MOSFET, respectively. Ib-type single crystalline diamond (001) substrate is boiled using a hotplate in a H_2_SO_4_ and HNO_3_ mixture solution at 300 °C for 3 h to clean surface contaminations (Figure 2ai,bI). The H-diamond channel layer is epitaxially grown on the cleaned substrate using microwave plasma-enhanced chemical vapor deposition technique (Figure 2aii,bII). Growth gases are H_2_ and CH_4_ with flow rates of 500 and 0.5 sccm, respectively. Chamber pressure is fixed at 80 Torr. Microwave power and growth temperature are in the range of 880~960 W and 900~940 °C, respectively. Thicknesses of the H-diamond epitaxial layers are in the range of 150~200 nm. Surface roughness confirmed by atomic force microscope is around 1.2 nm [45]. The sheet hole density and mobility investigated by Hall measurement are around 10^13^ cm^−2^ and 90 cm^2^·V^−1^·s^−1^, respectively. After growing the H-diamond channel layer, mesa-structure is formed for the H-diamond MOSFETs (Figure 2bIII) using capacitive coupled plasma dry-etching system. The chamber pressure and etching time are 10 Pa and 90 s, respectively. Etching gas (O_2_) flow rate and plasma power are 100 sccm and 50 W, respectively. It should be noted that there is no mesa-structure formation step for the fabrication of oxide/H-diamond MOS capacitor. High-*k* oxides of the ALD-Al_2_O_3_, ALD-HfO_2_, ALD-HfO_2_/ALD-Al_2_O_3_ multilayer, SD-HfO_2_/ALD-HfO_2_ bilayer, SD-TiO_2_/ALD-Al_2_O_3_ bilayer, and ALD-TiO_2_/ALD-Al_2_O_3_ bilayer are deposited as high-*k* oxides for the MOS capacitors and MOSFETs (Figure 2aiii,bIV). For the SD-HfO_2_/ALD-HfO_2_ and SD-TiO_2_/ALD-Al_2_O_3_ bilayers, the ALD-HfO_2_ and ALD-Al_2_O_3_ impact as buffer layers with thicknesses of around 4.0 nm to protect the hydrogen surface from being damaged by SD plasma discharge during the SD-HfO_2_ and SD-TiO_2_ depositions, respectively. For the ALD-TiO_2_/ALD-Al_2_O_3_ bilayer, the ALD-Al_2_O_3_ with thicknesses of 0~4.0 nm impacts as a buffer layer to suppress high leakage current density (*J*) of ALD-TiO_2_/H-diamond MOS capacitor due to a low valence band offset (*ΔE_V_*) between TiO_2_ and H-diamond [42]. The ALD-Al_2_O_3_, ALD-HfO_2_, and ALD-TiO_2_ are deposited using precursors of trimethylaluminum, tetrakis (ethylmethylamino) hafnium, and tetrakis(dimethylamino)titanium with water vapor, respectively. Although the high deposition temperature for the ALD-oxides can increase their properties possibly, we have deposited them at 120 °C. There are two reasons for the low deposition temperature. Since the hydrogen surface of the H-diamond is thermal sensitivity, the low deposition temperature can protect the surface from being damaged by the heating at high temperature. Additionally, since deposition areas of oxide insulators are patterned using LOR 5A/AZ 5214E bilayer photoresists and the temperature limitation of them is 150 °C, we set the temperature of 120 °C to deposit the ALD-oxides. The SD-HfO_2_ layer is deposited on the ALD-HfO_2_/H-diamond in a pure Ar ambient at RT. The radio-frequency (RF) power, gas flow rate, and chamber pressure are 30 W, 2.0 sccm, and 1 Pa, respectively. The SD-TiO_2_ layer is deposited on the ALD-Al_2_O_3_/H-diamond in an Ar+O_2_ ambient at RT. Oxygen content in the SD chamber is in the range of 0~20%. The RF power, total gas flow rate, and chamber pressure are 40 W, 2.0 sccm, and 1 Pa, respectively. Total thickness of oxide insulators for each MOS capacitor is in the range of 18.9~34.1 nm. After depositing the oxide insulators, gate cover metals of Au/Ti or Au/Ti/Pd are formed using evaporator system. Lastly, Au/Ti/Pd triple-layer metals are evaporated on the H-diamond for the source/drain ohmic contacts (Figure 2aiv,bV).

Band configurations of oxide/H-diamond heterointerfaces are determined using X-ray photoelectron spectroscopy (XPS) technique, which is performed with a monochromated Al K*α* X-ray source (*hv* = 1486.6 eV). All core level spectra are recorded with a 0.05 eV step and a 55 eV pass energy. Electrical properties of the MOS capacitors and MOSFETs are measured under a dark condition using MX-200/B prober and B1500A parameter analyzer at RT.

## 3. Results and Discussion

### 3.1. Band Configurations of High-k Oxide/H-Diamond Heterointerfaces

Band configurations of heterointerfaces are considered as the most fundamental properties in material physics. Understanding of them for high-*k* oxide/H-diamond heterojunctions is very important for the development of high-performance H-diamond-based MOS electronic devices. Band configurations of ALD-Al_2_O_3_/H-diamond [46], ALD-HfO_2_/H-diamond [46], and ALD-TiO_2_/ALD-Al_2_O_3_/H-diamond [31] are investigated. The *ΔE_V_* values of ALD-Al_2_O_3_/H-diamond and ALD-HfO_2_/H-diamond heterojunctions are calculated using the equation below,

(1) $$\Delta E_{V}={\left(E_{CL}-E_{VBM}\right)}_{H-dia.}-{\left(E_{CL}-E_{VBM}\right)}_{Oxide}^{Thick}-{\left(E_{CL}^{H-dia.}-\;E_{CL}^{oxide}\right)}_{Oxide}^{Thin}$$

where the ${\left(E_{CL}-E_{VBM}\right)}_{H-dia.}$ is the difference in binding energies between C 1*s* core level (CL) and valence band maximum (VBM) of the H-diamond. The ${\left(E_{CL}-E_{VBM}\right)}_{Oxide}^{Thick}$ is the difference in binding energies between Al 2*p*_3/2_ and VBM for the 20 nm thick Al_2_O_3_ sample or between Hf 4*f*_7/2_ and VBM for the 20 nm thick HfO_2_ sample. The ${\left(E_{CL}^{H-dia.}-E_{CL}^{oxide}\right)}_{Oxide}^{Thin}$ is the difference in binding energies between C 1*s* and Al 2*p*_3/2_ for the ALD-Al_2_O_3_ (4 nm)/H-diamond sample or between C 1*s* and Hf 4*f*_7/2_ for the ALD-HfO_2_ (4 nm)/H-diamond sample. The *ΔE_V_* for the ALD-TiO_2_/ALD-Al_2_O_3_ heterojunction can be calculated using the equation below,

(2) $$\Delta E_{V}={\left(E_{Ti2p_{3/2}}-E_{VBM}\right)}_{TiO_{2}}^{Thick}-{\left(E_{Al2p_{3/2}}-E_{VBM}\right)}_{Al_{2}O_{3}}^{Thick}-{\left(E_{Ti2p_{3/2}}-E_{Al2p_{3/2}}\right)}_{TiO_{2}}^{Thin}$$

where the ${\left(E_{Ti2p_{3/2}}-E_{VBM}\right)}_{TiO_{2}}^{Thick}$ is the difference in binding energies between Ti 2*p_3/2_* and VBM for the ALD-TiO_2_(25 nm)/ALD-Al_2_O_3_(4 nm) sample. The ${\left(E_{Al2p_{3/2}}-E_{VBM}\right)}_{Al_{2}O_{3}}^{Thick}$ is the difference in binding energies between Al 2*p*_3/2_ and VBM for the 20 nm thick Al_2_O_3_ film [46]. The ${\left(E_{Ti2p_{3/2}}-E_{Al2p_{3/2}}\right)}_{TiO_{2}}^{Thin}$ is the difference in binding energies between Ti 2*p_3/2_* and Al 2*p*_3/2_ for the ALD-TiO_2_ (3 nm)/ALD-Al_2_O_3_ (4 nm) sample. Since calculations of the *ΔE_V_* for the ALD-Al_2_O_3_/H-diamond, ALD-HfO_2_/H-diamond, and ALD-TiO_2_/ALD-Al_2_O_3_ heterojunctions are based on the relative energies for the two peaks of each sample, there are no charge-up effects for the deduced *ΔE_V_* values.

**Figure 3:**
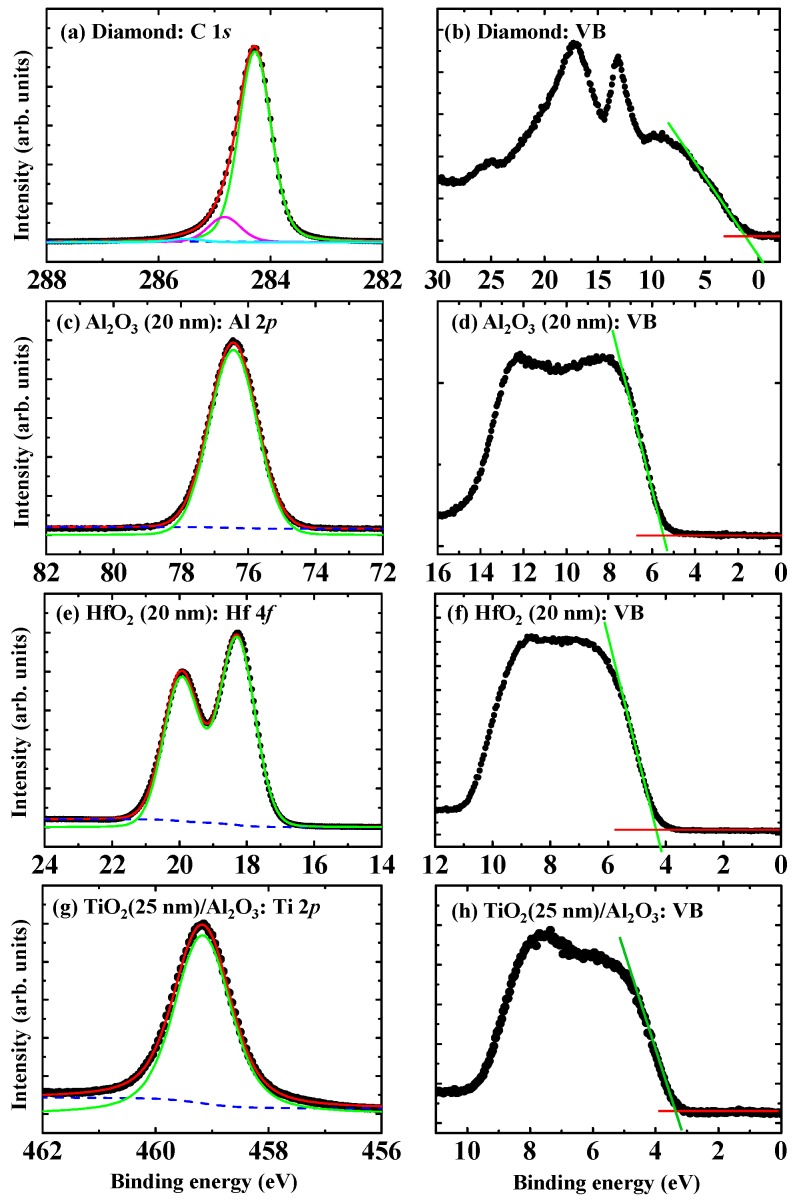
(**a**) C 1*s*, (**c**) Al 2*p*, (**e**) Hf 4*f*, and (**g**) Ti 2*p* photoelectron spectra for the H-diamond, Al_2_O_3_ (20 nm), HfO_2_ (20 nm), and TiO_2_ (25 nm)/Al_2_O_3_ (4 nm) samples, respectively. Valence band spectra for them are also shown in Figure 3b,d,f,h, respectively (Reprinted from references [31,46]).

Figure 3 shows CL and VB spectra for the H-diamond substrate (Figure 3a,b), ALD-Al_2_O_3_ (20 nm) (Figure 3c,d), ALD-HfO_2_ (20 nm) (Figure 3e,f), and ALD-TiO_2_ (25 nm)/ALD-Al_2_O_3_ (4 nm) (Figure 3g,h) measured by XPS technique. All the CL peaks are fitted using Voigt (mixed Lorentzian-Gaussian) line shapes and Shirley background. The valence band maxima of the H-diamond and thick oxide insulators are determined by extrapolating linear fitting to leading edges of the VB spectra to intersect with the baselines. The C 1*s* spectrum of H-diamond [Figure 3a] is fitted with three components of C-C, CH_x_, and C-OH [47]. Binding energy difference between C-C and CH_x_ is 0.6 eV. That between C-C and C-OH is 1.2 eV [48]. According to angle-resolved XPS measurement result [49,50], the CH_x_ and C-OH are attributed to surface contaminations. The Al-O (Figure 3c) and Hf-O [Figure 3e] are used to fit the Al 2*p* and Hf 4*f* spectra, respectively. The VBM values for the H-diamond, Al_2_O_3_ (20 nm), HfO_2_ (20 nm), and ALD-TiO_2_ (25 nm)/ALD-Al_2_O_3_ samples are determined to be 1.2 ± 0.2, 5.4 ± 0.2, 4.3 ± 0.2 eV, and 3.4 ± 0.2 eV, respectively.

**Figure 4:**
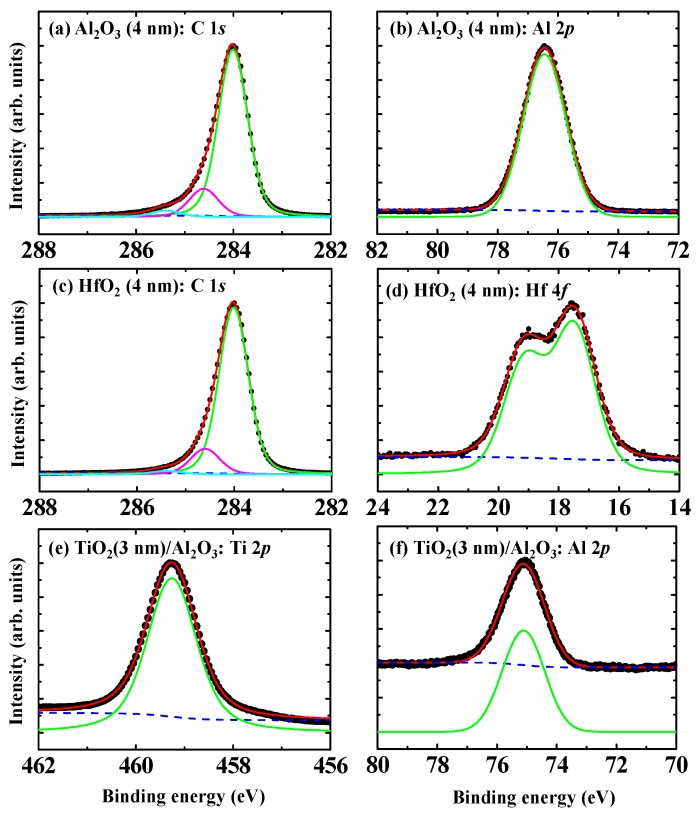
(**a**,**b**) C 1*s* and Al 2*p* for the Al_2_O_3_ (4 nm)/H-diamond, respectively; (**c**,**d**) C 1*s* and Hf 4*f* for the HfO_2_ (4 nm)/H-diamond, respectively; (**e**,**f**) Al 2*p* and Ti 2*p* spectra for the TiO_2_ (3 nm)/Al_2_O_3_ (4 nm)/H-diamond, respectively (Reprinted from references [31,46]).

Figure 4a,b show C 1*s* and Al 2*p* spectra for the Al_2_O_3_ (4 nm)/H-diamond sample, respectively. Figure 4c,d show C 1*s* and Hf 4*f* for the HfO_2_ (4 nm)/H-diamond sample, respectively. Figure 4e,f show Al 2*p* and Ti 2*p* spectra for TiO_2_ (3 nm)/Al_2_O_3_ (4 nm)/H-diamond sample, respectively. Three components of C-C, CH_x_, and C-OH are used to fit each C 1*s* spectrum, which is similar to those of the H-diamond substrate. The Al-O, Hf-O, and Ti-O are used to fit the Al 2*p*, Hf 4*f*, and Ti 2*p* spectra, respectively. Table 2 summarizes the CL peak energies and VBM values corresponding to the spectra in Figure 3 and Figure 4. The peak energies for the C 1*s* are relative to the C-C bonds. Binding energy error for each peak is ±0.2 V. By inserting CL binding energies and the VBM values into the Equations (1) and (2), the *ΔE_V_* values for the ALD-Al_2_O_3_/H-diamond, ALD-HfO_2_/H-diamond, and ALD-TiO_2_/ALD-Al_2_O_3_ heterojunctions are calculated to be 2.9 ± 0.2, 2.6 ± 0.2, and −0.6 ± 0.2 eV, respectively. Based on the bandgap energies of ALD-Al_2_O_3_ (7.2 eV) [46], ALD-HfO_2_ (5.4 eV) [46], and ALD-TiO_2_ (3.4 eV) [51], conduction band offset (*ΔE_C_*) values for them can be deduced to be 1.2 ± 0.2, 2.7 ± 0.2, and 3.2 ± 0.2 eV, respectively.

**Figure 5:**
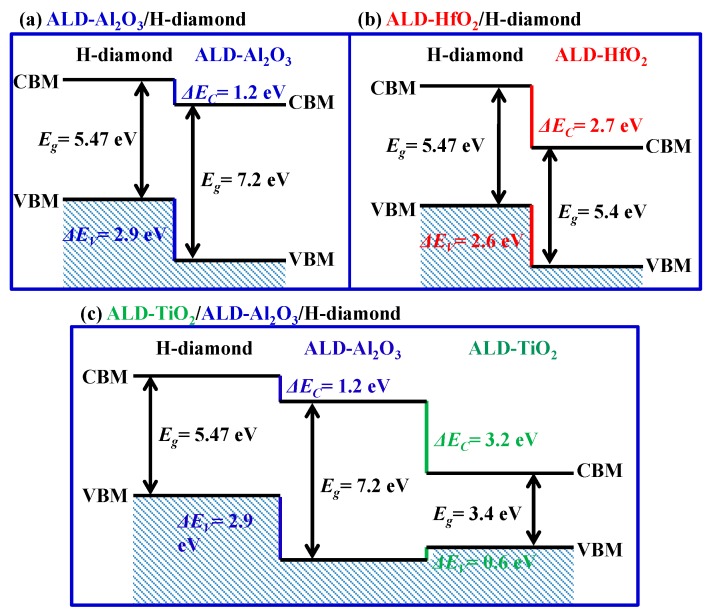
Schematic band configurations for (**a**) ALD-Al_2_O_3_/H-diamond, (**b**) ALD-HfO_2_/H-diamond, and (**c**) ALD-TiO_2_/ALD-Al_2_O_3_/H-diamond heterojunctions, respectively (Reprinted from references [31,46]).

Figure 5a–c show band configurations of the ALD-Al_2_O_3_/H-diamond, ALD-HfO_2_/H-diamond, and ALD-TiO_2_/ALD-Al_2_O_3_/H-diamond heterojunctions, respectively. There are type II staggering-type structures for the ALD-Al_2_O_3_/H-diamond and ALD-HfO_2_/H-diamond heterojunctions and a type I straddling-type structure for the ALD-TiO_2_/ALD-Al_2_O_3_ heterojunction, respectively. The *ΔE_V_* between ALD-TiO_2_ and H-diamond is deduced to be 2.3 ± 0.2 eV. Because there are very large *ΔE_V_* values between the H-diamond with ALD-Al_2_O_3_, ALD-HfO_2_, and ALD-TiO_2_/ALD-Al_2_O_3_, they are promising to fabricate MOS electronic devices with low leakage current densities.

### 3.2. High-k Oxides on H-Diamond for MOS Capacitors

#### 3.2.1. ALD-Al_2_O_3_ and ALD-HfO_2_ Single Layers

**Figure 6:**
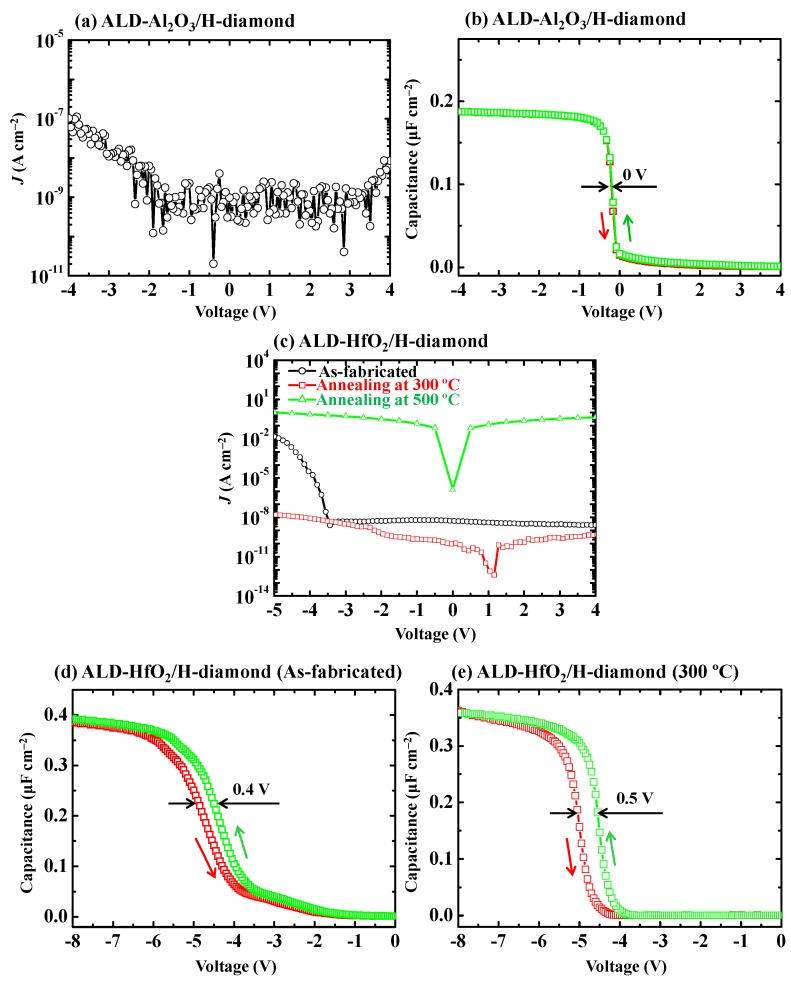
(**a**,**b**) *J*-V and *C*-*V* characteristics for the ALD-Al_2_O_3_/H-diamond MOS capacitor, respectively (Reprinted from reference [28]); (**c**) Annealing effect on *J*-V characteristics of ALD-HfO_2_/H-diamond MOS capacitors (Reprinted from reference [29]); (**d**,**e**) *C*-*V* characteristics of the ALD-HfO_2_/H-diamond MOS capacitors before and after annealing at 300 °C, respectively (Reprinted from reference [29]).

Since Al_2_O_3_ and HfO_2_ are two common high-*k* oxide insulators, electrical properties of ALD-Al_2_O_3_/H-diamond and ALD-HfO_2_/H-diamond MOS capacitors have been investigated firstly [28,29]. Figure 6a,b show *J*-V and *C*-*V* characteristics for ALD-Al_2_O_3_/H-diamond MOS capacitor, respectively. The *J* is deduced using leakage current divided by the gate electrode area. It is lower than 1.0 × 10^−7^ A·cm^−2^ as the gate voltage in the range of −4.0~4.0 V for the ALD-Al_2_O_3_/H-diamond MOS capacitor. Red and green lines in Figure 6b represent the *C*-*V* curves with the gate voltage sweeping directions from negative to positive and from positive to negative, respectively. Distinct accumulation region and sharp dependence at depletion region are observed in the *C*-*V* curves. Interfacial trapped charge density for the ALD-Al_2_O_3_/H-diamond is thus quite low [52]. There is a quite low voltage shift relative to 0 V in the depletion region for the *C*-*V* curves, indicating the low fixed charge density in the ALD-Al_2_O_3_ [53]. Hysteresis loop voltage for the *C*-*V* curves with the change of sweeping directions is 0 V, which suggests that there is low trapped charge density in the ALD-Al_2_O_3_ single layer. High quality Al_2_O_3_ film is thus deposited by the ALD technique at 120 °C. Maximum capacitance (*C_max_*) for the MOS capacitor is 0.187 μF·cm^−2^. By considering the ALD-Al_2_O_3_ thickness of 25.4 nm, the *k* value of the single ALD-Al_2_O_3_ layer can be calculated to be 5.4, which is much lower than that for the ideal Al_2_O_3_ of 8.5~9. This is possibly attributed to the low deposition temperature (120 °C) for our ALD-Al_2_O_3_ film.

Figure 6c shows annealing effect on the *J*-V characteristics of the ALD-HfO_2_/H-diamond MOS capacitors. For gate voltage higher than 0 V, the leakage current densities of the MOS capacitors before and after annealing at 300 °C are lower than 10^−8^ A·cm^−2^. For gate voltage more negative than 0 V, the *J* of MOS capacitor before annealing increases from 10^−8^ A·cm^−2^ at −3.4 V to 10^−2^ A·cm^−2^ at −5.0 V. However, the *J* after annealing at 300 °C is still lower than 1.6 × 10^−8^ A·cm^−2^. Therefore, after annealing at 300 °C, the qualities of the ALD-HfO_2_ film and the ALD-HfO_2_/H-diamond interface are improved greatly. When the annealing temperature increases to 500 °C, the large *J* is observed at gate voltage from −5.0 to 4.0 V, which is probably attributed to the formation of polycrystalline HfO_2_ film at this high temperature [54]. Figure 6d,e show *C*-*V* characteristics of ALD-HfO_2_/H-diamond MOS capacitors before and after annealing at 300 °C, respectively. Both *C*-*V* curves shift to the left hand sides greatly relative to 0 V, thus positive fixed charges with high densities exist in the bulk HfO_2_ film or close to the HfO_2_/H-diamond interface [53]. Both *C*-*V* curves show hysteresis loops with voltages of 0.4 and 0.5 V, respectively, which implies that there are higher trapped charge density in the bulk HfO_2_ film than that in the ALD-Al_2_O_3_ film, which is possibly ascribed to the oxygen vacancies in the ALD-HfO_2_ film. Noted that the *C*-*V* curves in the depletion regions for the ALD-HfO_2_/H-diamond MOS capacitor after annealing are sharper than those of before annealing and there is no residual capacitance from −1.2 V to −3.8 V in Figure 6e. Therefore, there is a lower interface trapped charge density for the ALD-HfO_2_/H-diamond interface after annealing than that before annealing [52]. The *C_max_* values for both MOS capacitors are 0.393 and 0.359 μF·cm^−2^, respectively. The difference is possibly ascribed to a little variation of gate electrode area during the fabrication. Based on the *C_max_* values and the HfO_2_ thickness (27.3 nm), the dielectric constants of the HfO_2_ films before and after annealing are calculated to be 12.1 and 11.2, respectively, which are in good agreement with the reported values (11.7~14) of ALD-HfO_2_ on Si and GaN substrates [54,55]. Low dielectric constants comparing with the ideal value of around 24 are believed to be the intrinsic property for the amorphous ALD-HfO_2_ deposited at low temperature (120 °C).

#### 3.2.2. ALD-HfO_2_/ALD-Al_2_O_3_ Multilayer and SD-HfO_2_/ALD-HfO_2_ Bilayer

**Figure 7:**
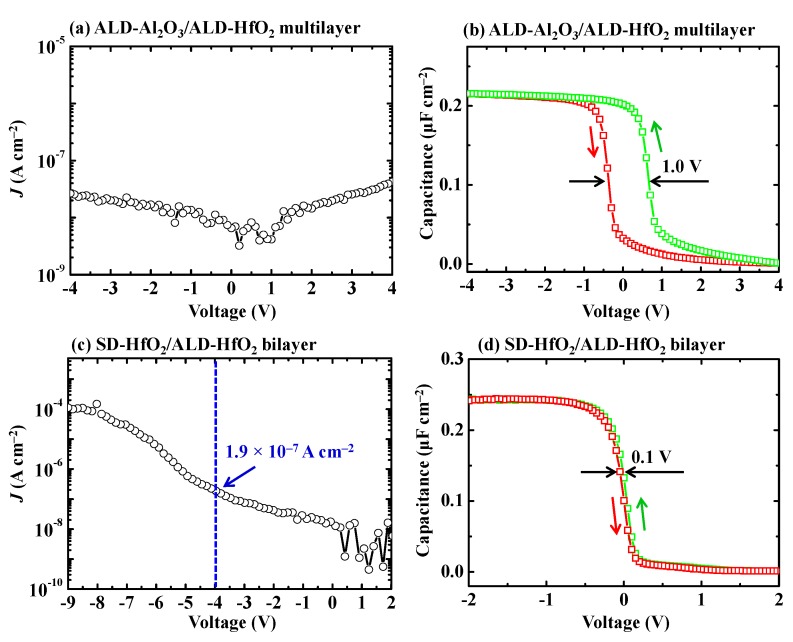
(**a**,**b**) *J*-V and *C*-*V* characteristics of the ALD-Al_2_O_3_/ALD-HfO_2_ multilayer on the H-diamond for MOS capacitor, respectively (Reprinted from reference [28]); (**c**,**d**) *J*-V and *C*-*V* characteristics for the SD-HfO_2_/ALD-HfO_2_ bilayer on the H-diamond for MOS capacitor, respectively (Reprinted from reference [30]).

Since *k* value of the ALD-Al_2_O_3_ single layer on the H-diamond is not high and there are high positive fixed charge densities in the ALD-HfO_2_, we investigate electrical properties of ALD-HfO_2_/ALD-Al_2_O_3_ multilayer and SD-HfO_2_/ALD-HfO_2_ bilayer on the H-diamond for MOS capacitors [28,30]. Figure 7a,b show *J*-V and *C*-*V* characteristics for the H-diamond MOS capacitor with the ALD-Al_2_O_3_/ALD-HfO_2_ multilayer as the oxide insulator, respectively. The ALD-Al_2_O_3_ and ALD-HfO_2_ are the first and top layers in contact with the H-diamond surface and gate cover metal, respectively. Each monolayer thickness for ALD-Al_2_O_3_ and ALD-HfO_2_ is 1.0 nm with total thickness for the multilayer of 32.0 nm. The *J* for the MOS capacitor in Figure 7a at −4.0 V is 2.7 × 10^−8^ A·cm^−2^, which is lower than those of ALD-Al_2_O_3_/H-diamond and as-fabricated ALD-HfO_2_/H-diamond MOS capacitors. The *C*-*V* curve of the MOS capacitor in Figure 7b shows decrease of stretch-out in the depletion region compared to that for the as-fabricated ALD-HfO_2_/H-diamond MOS capacitor. Therefore, the interfacial trapped charge densities for the MOS capacitor with the ALD-HfO_2_/ALD-Al_2_O_3_ multilayer as the oxide insulator are lower than that for the as-fabricated ALD-HfO_2_/H-diamond MOS capacitor [53]. However, there is a large hysteresis loop voltage of around 1.0 V for the *C*-*V* curves in Figure 7b, indicating the high trapped charge density in the ALD-HfO_2_/ALD-Al_2_O_3_ multilayer. The *C_max_* for the *C*-*V* curves of the MOS capacitor is 0.216 μF·cm^−2^ and the *k* value for the HfO_2_/Al_2_O_3_ multilayer can be calculated to be 7.8, which is lower than that for the ALD-HfO_2_ single layer of 12.1 and larger than that for the ALD-Al_2_O_3_ single layer of 5.4.

Figure 7c,d show *J*-V and *C*-*V* characteristics for the SD-HfO_2_/ALD-HfO_2_/H-diamond MOS capacitor. The *J* increases with the gate voltage changing from 0 to −9.0 V. It is 1.9 × 10^−7^ A·cm^−2^ at gate voltage of −4.0 V, which is close to that of the single ALD-Al_2_O_3_/H-diamond MOS capacitor and lower than that of as-fabricated ALD-HfO_2_/H-diamond MOS capacitor. The maximum *J* value at −9.0 V is 1.1 × 10^−4^ A·cm^−2^. Based on *C_max_* of 0.244 μF·cm^−2^ and the total SD-HfO_2_/ALD-HfO_2_ thickness (34.1 nm), the *k* of the SD-HfO_2_/ALD-HfO_2_ bilayer is calculated to be 9.4, which is smaller than the value (12.1) of the ALD-HfO_2_ single layer. This is probably attributed to the low deposition temperature (at RT) for the SD-HfO_2_. The voltage shift relative to 0 V of the *C*-*V* curves for the SD-HfO_2_/ALD-HfO_2_/H-diamond MOS capacitor is one order magnitude lower than that for the ALD-HfO_2_/H-diamond MOS capacitor. Thus, the issue of high fixed charge density for the ALD-HfO_2_/H-diamond MOS capacitor is resolved. Additionally, the *C*-*V* curve in the depletion region shows sharp dependence and small hysteresis voltage loop of 0.1 V for the SD-HfO_2_/ALD-HfO_2_/H-diamond MOS capacitor, indicating its low trapped charge densities in the SD-HfO_2_/ALD-HfO_2_ bilayer and at the SD-HfO_2_/ALD-HfO_2_/H-diamond interfaces.

#### 3.2.3. SD-TiO_2_/ALD-Al_2_O_3_ and ALD-TiO_2_/ALD-Al_2_O_3_ Bilayers

**Figure 8:**
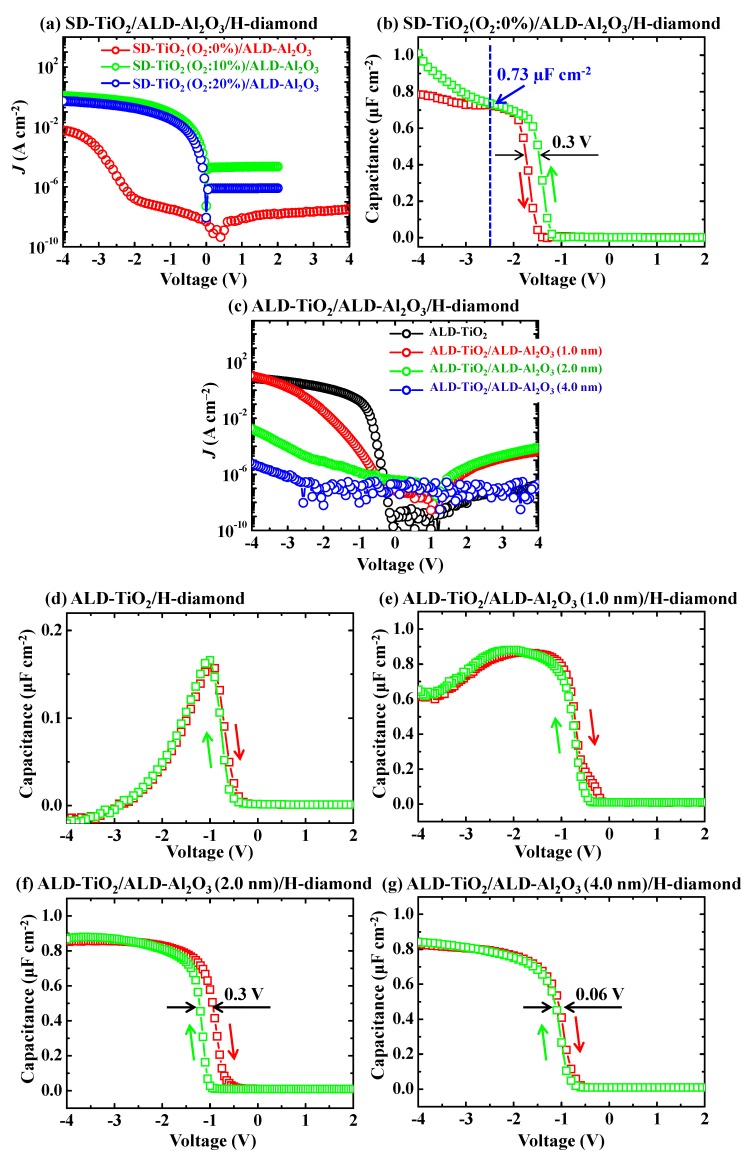
(**a**) *J*-V characteristics of the SD-TiO_2_/ALD-Al_2_O_3_/H-diamond MOS capacitors with O_2_ content in the SD chamber of 0%, 10%, and 20%, respectively (Reprinted from reference [31]); (**b**) *C*-*V* characteristics of the SD-TiO_2_ (O_2_: 0%)/ALD-Al_2_O_3_/H-diamond MOS capacitor; (**c**) *J*-V characteristics of ALD-TiO_2_/ALD-Al_2_O_3_/H-diamond MOS capacitors with ALD-Al_2_O_3_ buffer layer thicknesses of 0, 1.0, 2.0, and 4.0 nm, respectively; (**d**–**g**) *C*-*V* characteristics of the ALD-TiO_2_/ALD-Al_2_O_3_/H-diamond MOS capacitors with ALD-Al_2_O_3_ buffer layer thicknesses of 0, 1.0, 2.0, and 4.0 nm, respectively (Reprinted from reference [31]).

Figure 8a shows *J*-V characteristics for the SD-TiO_2_/ALD-Al_2_O_3_/H-diamond MOS capacitors. Thicknesses of SD-TiO_2_ films are 24.4, 18.9, and 23.3 nm with the change of oxygen gas content in the SD chamber of 0%, 10%, and 20%, respectively. When the chamber gas during the SD-TiO_2_ deposition is only Ar (O_2_: 0%), the *J* of the received MOS capacitor is lower than 10^−7^ A·cm^−2^ with the gate voltage changing from −2.0 to 4.0 V. As the gate voltage sweeps from −2.0 to −4.0 V, the *J* of the MOS capacitor increases from 10^−7^ to as large as 10^−2^ A·cm^−2^. As the oxygen gas content in the SD chamber increases to 10% and 20%, there are very high leakage current densities for the MOS capacitors. Figure 8b shows the *C*-*V* characteristic of the SD-TiO_2_/ALD-Al_2_O_3_/H-diamond MOS capacitor with 0% O_2_ content in the SD chamber during the SD-TiO_2_ deposition. Depletion regions of the *C*-*V* curves locate at the left hand side relative to 0 V. Thus, positive charges exist at the ALD-Al_2_O_3_/H-diamond interface or in the SD-TiO_2_/ALD-Al_2_O_3_ bilayer. It is clarified above that there are rarely positive charges for the ALD-Al_2_O_3_/H-diamond MOS capacitor. Therefore, the positive charges in the SD-TiO_2_ (O_2_: 0%)/ALD-Al_2_O_3_/H-diamond MOS capacitor possibly exist in the SD-TiO_2_/ALD-Al_2_O_3_ bilayer. When the gate voltage shifts to the left hand side relative to −2.5 V (blue dashed line), the capacitance maxima separate with the gate voltage sweeping directions, which is possibly attributed to the high *J* at voltage of −4.0~−2.5 V. A hysteresis loop (0.3 V) for the *C*-*V* curve is possibly ascribed to the existence of trapped charges in the SD-TiO_2_/ALD-Al_2_O_3_ bilayer. The *C_max_* for the SD-TiO_2_ (O_2_: 0%)/ALD-Al_2_O_3_/H-diamond MOS capacitor is 0.73 µF cm^−2^ at −2.5 V. The *k* value of the SD-TiO_2_/ALD-Al_2_O_3_ bilayer can be calculated to be 22.5.

Figure 8c shows the leakage current densities of the ALD-TiO_2_/ALD-Al_2_O_3_/H-diamond MOS capacitors. The ALD-TiO_2_ thickness for each sample is 25.0 nm. The ALD-Al_2_O_3_ buffer layer thicknesses are 0, 1.0, 2.0, and 4.0 nm, respectively. The black, red, green, and blue lines represent the *J* for the MOS capacitors with the ALD-Al_2_O_3_ buffer layer thickness changing from 0 to 4.0 nm, respectively. With increase of the ALD-Al_2_O_3_ buffer layer thickness, the *J* for the MOS capacitors decreases at gate voltage of −4.0 V. Since the *∆E_V_* at the ALD-TiO_2_/H-diamond heterointerface is low, the *J* for the ALD-TiO_2_/H-diamond MOS capacitor is quite high [41]. While there is 1.0 nm-thick Al_2_O_3_ buffer layer for the ALD-TiO_2_/ALD-Al_2_O_3_/H-diamond MOS capacitor, the *J* of it is still high due to hole tunneling effect. When the buffer layer thicknesses are 2.0 and 4.0 nm, the leakage current densities of the MOS capacitors are improved to be lower than 1.7 × 10^−3^ and 6.0 × 10^−6^ A·cm^−2^, respectively. Figure 8d–g show the *C*-*V* characteristics for ALD-TiO_2_/ALD-Al_2_O_3_/H-diamond MOS capacitors with ALD-Al_2_O_3_ buffer layer thicknesses of 0, 1.0, 2.0, and 4.0 nm, respectively. The depletion regions for all the *C*-*V* curves locate at left hand sides relative to 0 V. Thus, there are positive charges at the ALD-TiO_2_/ALD-Al_2_O_3_ bilayers [53]. In Figure 8d,e, the *C_max_* values decrease greatly with the gate voltages changing from −1.0 to −4.0 V and from −2.0 to −4.0 V, respectively, which are possibly attributed to their high leakage current densities. When the thickness of ALD-Al_2_O_3_ buffer layer increases to be 2.0 nm, there are distinct accumulation and depletion regions for the C-V characteristics of the MOS capacitor. However, a small hysteresis loop of 0.3 V exists. On the contrary, the hysteresis loop is only 0.06 V for the MOS capacitor with the ALD-Al_2_O_3_ buffer layer thickness of 4.0 nm. According to the *C_max_* value of 0.83 µF·cm^−2^ for the ALD-TiO_2_ (25.0 nm)/ALD-Al_2_O_3_ (4.0 nm)/H-diamond MOS capacitor, the *k* value for the ALD-TiO_2_/ALD-Al_2_O_3_ bilayer is deduced to be 27.2, which is larger than those of other oxide insulators on the H-diamond for the MOS capacitors.

#### 3.2.4. Discussion for High-*k* Oxide/H-Diamond MOS Capacitors

We have demonstrated above for the electronic properties of several high-*k* oxide insulators on the H-diamond for the MOS capacitors. The *J* at −4.0 V, *k* values of oxide, C-V curve hysteresis loop voltage, and C-V curve voltage shift relative to 0 V for them are summarized in Table 3. Except for SD-TiO_2_ (O_2_: 0%)/ALD-Al_2_O_3_/H-diamond MOS capacitor, the *J* at −4.0 V for other MOS capacitors is lower than 6.0 × 10^−6^ A·cm^−2^. The *k* value of ALD-TiO_2_/ALD-Al_2_O_3_ bilayer (27.2) is higher than those of ALD-Al_2_O_3_ (5.4), ALD-HfO_2_ (12.1), ALD-HfO_2_/ALD-Al_2_O_3_ multilayer (7.8), SD-HfO_2_/ALD-HfO_2_ bilayer (9.1), and SD-TiO_2_/ALD-Al_2_O_3_ bilayer (22.5). The hysteresis loop voltages for C-V curves of the ALD-Al_2_O_3_/H-diamond, SD-HfO_2_/ALD-HfO_2_/H-diamond, and SD-TiO_2_/ALD-Al_2_O_3_/H-diamond MOS capacitors are lower than 0.1 V, indicating their low trapped charge densities in the oxide insulators. There are large voltage shift relative to 0 V for the ALD-HfO_2_/H-diamond, SD-TiO_2_/ALD-Al_2_O_3_/H-diamond, and ALD-TiO_2_/ALD-Al_2_O_3_/H-diamond MOS capacitors. Since hysteresis loop of C-V curves for the MOS capacitor with ALD-HfO_2_/ALD-Al_2_O_3_ multilayer as oxide insulator is very large, we do not show its voltage shift relative to 0 V. There are small voltage shifts for the ALD-Al_2_O_3_/H-diamond and SD-HfO_2_/ALD-HfO_2_/H-diamond MOS capacitors, indicating their low fixed charged densities in the oxides. For the MOS capacitor gas sensors, reactions between gases and catalytic metals make charges accumulation at the metal/oxide interfaces, leading to the shift of C-V curve in the deletion region. Therefore, the best oxide insulators for the MOS capacitor gas sensors are those having low fixed and trapped charge densities. Based on the electrical properties of MOS capacitors, the ALD-Al_2_O_3_ and SD-HfO_2_/ALD-HfO_2_ bilayer are possibly two good choices for the applications of the H-diamond MOS capacitor gas sensors.

### 3.3. Electrical Properties of H-Diamond MOSFETs

**Figure 9:**
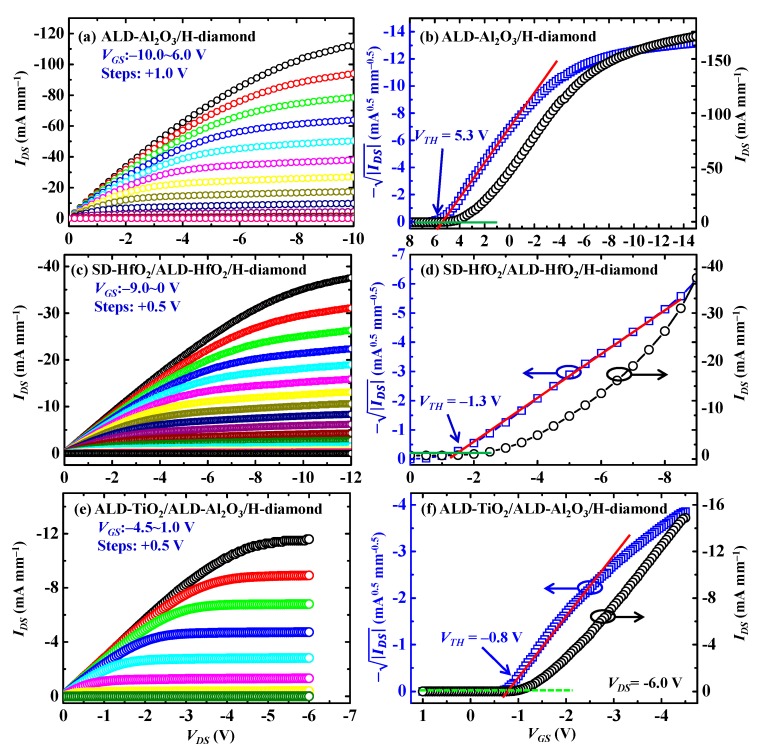
(**a**) *I_DS_*-*V_DS_* and (**b**) $-\sqrt{\left|I_{DS}\right|}$-*V_GS_* characteristics for the ALD-Al_2_O_3_/H-diamond MOSFET, respectively; (**c**) *I_DS_*-*V_DS_* and (**d**) $-\sqrt{\left|I_{DS}\right|}$ -*V_GS_* characteristics for the SD-HfO_2_/ALD-HfO_2_/H-diamond MOSFET, respectively; (**e**) *I_DS_*-*V_DS_* and (**f**) $-\sqrt{\left|I_{DS}\right|}$ -*V_GS_* characteristics for the ALD-TiO_2_/ALD-Al_2_O_3_/H-diamond MOSFET, respectively (Reprinted from references [30,31,39]).

Since there are low fixed and trapped charge densities for the ALD-Al_2_O_3_/H-diamond and SD-HfO_2_/ALD-HfO_2_/H-diamond MOS capacitors and there is the highest *k* value for the ALD-TiO_2_/ALD-Al_2_O_3_ bilayer, we will show electrical properties of the ALD-Al_2_O_3_/H-diamond, SD-HfO_2_/ALD-HfO_2_/H-diamond, and ALD-TiO_2_/ALD-Al_2_O_3_/H-diamond MOSFETs. Figure 9a,c,e show drain-source current versus drain voltage (*I_DS_*-*V_DS_*) characteristics for the ALD-Al_2_O_3_/H-diamond, SD-HfO_2_/ALD-HfO_2_/H-diamond, and ALD-TiO_2_/ALD-Al_2_O_3_/H-diamond MOSFETs, respectively. Gate length (*L_G_*) and gate width (*W_G_*) are 3 and 100 μm for the ALD-Al_2_O_3_/H-diamond MOSFET, respectively [39]. Those are 4 and 150 μm for the SD-HfO_2_/ALD-HfO_2_/H-diamond and ALD-TiO_2_/ALD-Al_2_O_3_/H-diamond MOSFETs, respectively [30]. Interspaces between source/drain and gate are around 1.2 ± 0.1, 5.0, and 4.0 μm for the ALD-Al_2_O_3_/H-diamond, SD-HfO_2_/ALD-HfO_2_/H-diamond, and ALD-TiO_2_/ALD-Al_2_O_3_/H-diamond MOSFETs, respectively. Gate-source voltage (*V_GS_*) is varied from −10.0 to 6.0 V in steps of +1.0 V for the ALD-Al_2_O_3_/H-diamond MOSFET. Those for the SD-HfO_2_/ALD-HfO_2_/H-diamond and ALD-TiO_2_/ALD-Al_2_O_3_/H-diamond MOSFETs are varied from −9.0 to 0 V in steps of +0.5 V and from −4.5 to 1.0 V in steps of +0.5 V, respectively. All of curves show obvious pinch-off and *p*-type characteristics. The maximum *I_DS_* (*I_DSmax_*) values are −112.4, −37.6, and −11.6 mA·mm^−1^, respectively. Difference of *I_DSmax_* for the three MOSFETs is attributed to the variations of H-diamond channel layer hole density and MOSFET device structures referring to *L_G_*, *W_G_*, and interspaces between source/drain and gate electrodes. Figure 9b,d,f show $-\sqrt{\left|I_{DS}\right|}$-*V_GS_* characteristics for the ALD-Al_2_O_3_/H-diamond, SD-HfO_2_/ALD-HfO_2_/H-diamond, and ALD-TiO_2_/ALD-Al_2_O_3_/H-diamond MOSFETs, respectively. The *V_TH_* values of them are 5.3 ± 0.1, −1.3 ± 0.1 V, and −0.8 ± 0.1, respectively. Thus, the ALD-Al_2_O_3_/H-diamond MOSFET operate with a depletion-mode characteristic. The SD-HfO_2_/ALD-HfO_2_/H-diamond and ALD-TiO_2_/ALD-Al_2_O_3_/H-diamond MOSFETs operate with enhancement-mode characteristics, which indicate that there is no current output at *V_GS_* = 0 V. Hole accumulation conditions for the H-diamond channel layer are surface carbon-hydrogen bonds and negatively adsorbed layer [56,57]. Since the carbon-hydrogen bonds are very stable at the temperature lower than 250 °C, the enhancement-mode characteristics for the MOSFETs result from the disappearance of negatively adsorbed layer or the formation of positive charges at the SD-HfO_2_/ALD-HfO_2_/H-diamond and ALD-TiO_2_/ALD-Al_2_O_3_/H-diamond interfaces [58]. On/off ratios for all MOSFETs are higher than 10^8^. Subthreshold swings are 138, 195, and 79 mV·dec^−1^ for the ALD-Al_2_O_3_/H-diamond, SD-HfO_2_/ALD-HfO_2_/H-diamond, and ALD-TiO_2_/ALD-Al_2_O_3_/H-diamond MOSFETs, respectively [31,39].

There is the following relationship between on-resistance (*R_ON_*) and effective mobility (*μ_eff_*) for the H-diamond channel layer of the MOSFETs with condition of *R_ON_* and source/drain-to-gate resistance (2*R_SD_*) much higher than source/drain ohmic contact resistances.

(3) $$R_{ON}=R_{CH}+2R_{SD}={\left[{\left(\frac{\partial I_{DS}}{\partial V_{DS}}\right)}_{V_{DS}=0}\right]}^{-1}=\frac{L_{G}}{W_{G}\times \mu_{eff}\times C_{OX}\times (V_{GS}-V_{TH})}+2R_{SD}$$

where the *R_ON_* normalized by the *W_G_* was obtained from fitting the *I_DS_-V_DS_* curve. The *C_OX_* is the *C_max_* of the oxide insulator. The 2*R_SD_* value was determined based on the linear function between the *L_G_* and *R_ON_*. Since the 2*R_SD_* values for the ALD-Al_2_O_3_/H-diamond and ALD-TiO_2_/ALD-Al_2_O_3_/H-diamond MOSFETs were not investigated in our previous studies [31,39], we only calculate the *μ_eff_* of H-diamond channel layer for the SD-HfO_2_/ALD-HfO_2_/H-diamond MOSFET [30], which is around 38.7 ± 0.5 cm^2^·V^−1^·s^−1^.

## 4. Conclusions

Here, fabrication routines for the H-diamond MOS capacitor and MOSFET, band configurations of high-*k* oxide/H-diamond heterointerfaces, and electrical properties of the H-diamond MOS capacitors and MOSFETs were summarized. There were high valence band offsets for ALD-Al_2_O_3_/H-diamond, ALD-HfO_2_/H-diamond, and ALD-TiO_2_/ALD-Al_2_O_3_/H-diamond heterointerfaces. Electrical properties of the H-diamond MOS capacitors with the ALD-Al_2_O_3_, ALD-HfO_2_, ALD-HfO_2_/ALD-Al_2_O_3_ multilayer, SD-HfO_2_/ALD-HfO_2_ bilayer, SD-TiO_2_/ALD-Al_2_O_3_ bilayer, and ALD-TiO_2_/ALD-Al_2_O_3_ bilayer were investigated and discussed. Except for the SD-TiO_2_/ALD-Al_2_O_3_/H-diamond MOS capacitor, the leakage current densities at −4.0 V for the other MOS capacitors were lower than 6.0 × 10^−6^ A·cm^−2^. The *k* value of ALD-TiO_2_/ALD-Al_2_O_3_ bilayer was 27.2, which was higher than those of other oxide insulators. There were low fixed and trapped charge densities for the ALD-Al_2_O_3_/H-diamond and SD-HfO_2_/ALD-HfO_2_/H-diamond MOS capacitors and good operations for the MOSFETs. These characteristics made them promising for the fabrication of high-performance H-diamond MOS capacitor and MOSFET gas sensors.

## Figures and Tables

**Table 1:** Material properties of Si, 4H-SiC, GaN, and diamond at room temperature [4,5,6].

Properties	Si	4H-SiC	GaN	Diamond
Bandgap energy (eV)	1.12	3.2	3.4	5.47
Breakdown field (MV·cm^−1^)	0.3	3	5	10
Thermal conductivity (W·cm^−1^·K^−1^)	1.5	5.0	1.3	24
Electron mobility (cm^2^·V^−1^·s^−1^)	1450	900	2000	4500
Hole mobility (cm^2^·V^−1^·s^−1^)	480	120	200	3800
Saturation electron velocity (×10^7^ cm^−1^)	0.86	3	2.5	2
Saturation hole velocity (×10^7^ cm^−1^)	-	-	-	0.8

**Table 2:** The binding energies (eV) of C 1*s*, Al 2*p*_3/2_, Hf 4*f*_7/2_, Ti 2*p*_3/2_, and VBM for the H-diamond and oxide insulators corresponding to the peaks in Figure 3 and Figure 4 [31,46].

Sample	C 1s	Al 2*p*_3/2_	Hf 4*f*_7/2_	Ti 2*p*_3/2_	VBM
H-diamond	284.3				1.2
Al_2_O_3_ (20 nm)		76.3			5.4
Al_2_O_3_ (4 nm)	284.0	74.7			
HfO_2_ (20 nm)			18.3		4.3
HfO_2_ (4 nm)	284.0		17.5		
TiO_2_ (25 nm)/Al_2_O_3_				459.2	3.4
TiO_2_ (3 nm)/Al_2_O_3_		75.0		459.3	

**Table 3:** Electrical properties of high-*k* oxide/H-diamond MOS capacitors.

Oxide Insulators	*J* at −4.0 V (A·cm^−2^)	*k*	Hysteresis Loop Voltage (V)	Voltage Shift Related to 0 V (V)
ALD-Al_2_O_3_	1.0 × 10^−7^	5.4	0	small
ALD-HfO_2_ (300 °C annealing)	8.5 × 10^−9^	11.2	0.5	large
ALD-HfO_2_/ALD-Al_2_O_3_ multilayer	2.7 × 10^−8^	7.6	1.0	-
SD-HfO_2_/ALD-HfO_2_ bilayer	1.9 × 10^−7^	9.1	0.1	small
SD-TiO_2_ (O_2_: 0%)/ALD-Al_2_O_3_ bilayer	1.0 × 10^−2^	22.5	0.3	large
ALD-TiO_2_/ALD-Al_2_O_3_ (4 nm) bilayer	6.0 × 10^−6^	27.2	0.06	large
